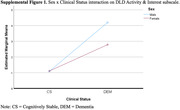# Sex Differences in Neuropsychiatric Symptoms Associated with Alzheimer’s Disease in Down Syndrome

**DOI:** 10.1002/alz.091071

**Published:** 2025-01-03

**Authors:** Sophia E. Shaka, Sharon J. Krinsky‐McHale, Wayne Silverman, Margaret B. Pulsifer, H. Diana Rosas, Florence Lai, Ira T. Lott, Christy L. Hom

**Affiliations:** ^1^ University of California, Irvine, Irvine, CA USA; ^2^ New York State Institute for Basic Research in Developmental Disabilities, Staten Island, NY USA; ^3^ Massachusetts General Hospital, Harvard Medical School, Boston, MA USA

## Abstract

**Background:**

Adults with Down syndrome (DS) have increased risk for developing Alzheimer’s disease (AD). Several studies have found that the onset of new neuropsychiatric and behavioral symptoms in DS is associated with AD progression. There is evidence in the neurotypical population that AD‐related apathy is more severe among males, while females display higher prevalence of depression, psychosis, and delusions. The current study investigates sex differences in AD‐related neuropsychiatric symptomology in adults with DS. It is hypothesized that men will have greater frequency or severity of AD‐related apathy compared to women while women will display more AD‐related depression.

**Method:**

A total of 155 adults with DS from the Alzheimer’s Disease in Down Syndrome (ADDS) study (43.3% female, M_age_ = 50.26, SD = 7.20) received cognitive testing. Their care providers completed questionnaires that measured psychiatric and/or behavioral symptoms: the Neuropsychiatric Inventory Questionnaire (NPI‐Q), the Reiss Screen for Maladaptive Behavior (RSMB), and the Dementia Questionnaire for People with Learning Disabilities (DLD). AD clinical status was determined through consensus decisions by the research team based on cognitive and functional scores. 113 participants were classified as cognitively stable and 42 had possible/probable AD dementia. Participants with mild cognitive impairment or of uncertain clinical status were excluded.

**Result:**

There were no significant main effects for sex on any of the measures examined. However, there was a significant interaction between sex and clinical status on the DLD Activity & Interest scale, adjusting for age, sex, and clinical status (F(4, 154) = 4.055, p = 0.46, η^2^ = 0.20). While cognitively stable men and women had similar scores on this scale, men with dementia had significantly greater problems in this area of functioning than women with dementia.

**Conclusion:**

AD appears to affect men and women differently after the transition to dementia. Affected males engage in fewer activities and are less interested in their surroundings than females, suggesting greater apathy or withdrawal, despite the lack of sex differences in depressive symptomology during the preclinical and clinical stages of AD. Possible neurobiological reasons will be discussed as well as how individuals with DS and AD may need tailored support based on sex differences in psychiatric and behavioral symptoms.